# Quasi‐Solid‐State Aluminum–Air Batteries with Ultra‐high Energy Density and Uniform Aluminum Stripping Behavior

**DOI:** 10.1002/advs.202304214

**Published:** 2023-08-16

**Authors:** Chaonan Lv, Yixin Li, Yuanxin Zhu, Yuxin Zhang, Jialin Kuang, Qing Zhao, Yougen Tang, Haiyan Wang

**Affiliations:** ^1^ Hunan Provincial Key Laboratory of Chemical Power Sources College of Chemistry and Chemical Engineering Central South University Changsha 410083 P. R. China; ^2^ Key Laboratory of Advanced Energy Materials Chemistry (Ministry of Education) College of Chemistry Nankai University Tianjin 300071 P. R. China

**Keywords:** aluminum–air battery, clay, hydrogen evolution reaction, quasi‐solid‐state electrolyte

## Abstract

Aqueous aluminum–air batteries are attracting considerable attention with high theoretical capacity, low‐cost and high safety. However, lifespan and safety of the battery are still limited by the inevitable hydrogen evolution reaction on the metal aluminum anode and electrolyte leakage. Herein, for the first time, a clay‐based quasi‐solid‐state electrolyte is proposed to address such issues, which has excellent compatibility and a liquid‐like ionic conductivity. The clay with uniform pore channels facilitates aluminum ions uniform stripping and reduces the activity of free H_2_O molecules by reconstructing hydrogen bonds network, thus suppressing the self‐corrosion of aluminum anode. As a result, the fabricated aluminum–air battery achieves the highest energy density of 4.56 KWh kg^−1^ with liquid‐like operating voltage of 1.65 V and outstanding specific capacity of 2765 mAh g^−1^, superior to those reported aluminum–air batteries. The principle of constructing quasi‐solid‐state electrolyte using low‐cost clay may further promote the commercialization of aluminum–air batteries and provide a new insight into electrolyte design for aqueous energy storage system.

## Graphical abstract

This work provides a novel low‐cost quasi‐solid‐state electrolyte that greatly inhibits self‐corrosion of metal Al anode and promotes uniform Al ions stripping. As a result, the Al–air battery achieves ultra‐high energy density and stable operating voltage using this quasi‐solid‐state electrolyte.

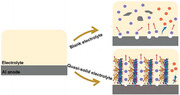

## 1 Introduction

**Figure 1:**
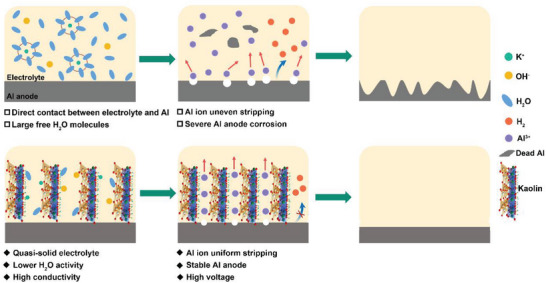
Schematic illustration of Al anode in Blank and quasi‐solid‐state electrolytes.

Aqueous aluminum–air (Al–air) batteries are the ideal candidates for the next generation energy storage/conversion system, owing to their high power and energy density (8.1 kWh kg^−1^), abundant resource (8.1 wt.% in Earth's crust), environmental friendliness.^[^ ^1^, ^2^, ^3^, ^4^, ^5^ ^]^ In addition, the discharge by‐product Al(OH)_3_ can be recycled and converted to metal Al after electrolysis, thus the efficient recycling of metal Al is realized. Actually, Al–air batteries have been successfully developed and applied in military communications, unmanned underwater/aerial vehicles. Especially, an Al–air battery weighing 100 kg was assembled and provided a range of over 3000 km for the electric vehicle.^[^ ^6^ ^]^ Unfortunately, their large‐scale commercial applications still remain serious challenges. Al–air batteries usually adopt strong alkaline solution (KOH/NaOH) as the electrolytes to remove the oxide layer on the surface of Al metal anode and dissolve the initial discharge by‐product and reduce the polarization of batteries. However, a series of notorious parasitic reactions such as self‐corrosion and hydrogen evolution reaction (HER) tend to occur on the Al metal surface due to the high activity of Al metal during the shelving and discharging process, accelerating metal corrosion and decreasing anode utilization.^[^ ^7^, ^8^, ^9^, ^10^ ^]^ Moreover, the stripping of Al ions during the discharge process is disordered due to the different activity and the existence of complex chemical reactions on the Al surface, resulting in the unstable discharge voltage and poor anode utilization (**Figure** **1**).^[^ ^11^, ^12^, ^13^ ^]^ As a result, it is highly desired to explore effective strategies to protect the Al anode and achieve uniform Al stripping behavior.

Scientifically, HER of Al metal in aqueous electrolyte is due to the decomposition of H_2_O molecules on the Al surface. To this end, the main strategies to address the above issues include increasing the hydrogen evolution overpotential of Al anode, reducing the direct contact between Al anode and electrolyte, and modifying the electrolyte structure. The overpotential of Al anode can be improved by making binary or multicomponent Al alloys, yet the manufacturing process of the alloy is cumbersome and alloy anode is not conducive to recycling of Al metal.^[^ ^14^, ^15^, ^16^ ^]^ Organic/inorganic hybrid additives, which can form a tight protective layer on the Al surface by electrochemical reduction and physical/chemical adsorption, are widely investigated owing to their simple operation.^[^ ^17^, ^18^ ^]^ This formed protective layer can not only prevent direct contact between the electrolyte and Al anode, but also cover the hydrogen evolution sites, thus suppressing Al corrosion.^[^ ^19^ ^]^ However, considering the high price and toxicity of additives, this strategy is unsuitable for large‐scale applications. Extensive investigations in electrolyte engineering modifications are enhancing the concentration of the electrolyte to reduce the content and activity of free H_2_O, including “water‐in‐salt” or highly concentrated organic additives, such as 16 m potassium acetate (KOAc),^[^ ^20^ ^]^ 40 vol% dimethyl sulfoxide (DMSO),^[^ ^21^ ^]^ 40 vol% glycerol (Gly) and 2 m sucrose.^[^ ^22^, ^23^, ^24^ ^]^ Although this strategy can effectively inhibit Al corrosion, the high viscosity, high cost, low ionic conductivity, potential safety risks of organic additives and seriously increased electrolyte weight hinder the actual applications of Al–air batteries. In addition to the HER, the penetration and leakage of liquid electrolyte through the capillaries in the porous air cathode and battery mold is another obstacle to further commercialization of Al–air battery.^[^ ^25^ ^]^ Solid state electrolytes including gel and polymer electrolytes can effectively solve the above issues, however, the previously reported solid state electrolytes have inferior ionic conductivity and cannot achieve high energy and power density.

Recently, functionalized kaolin clay as electrodes materials, separators and nanofillers have achieved more attention in the fields of energy storage/conversion owing to its low‐cost, abundant resources, fire resistance, and stable thermal.^[^ ^26^, ^27^, ^28^ ^]^ Furthermore, the porous structure and high surface area of kaolin allow it to have a large contact area with electrolyte, which provides abundant active sites (O─H, Si─O, and Al─O) and fast ion channels.^[^ ^29^ ^]^ In addition, the abundant hydrophilic groups on the clay surface can anchor H_2_O molecules by hydrogen bond or covalent bond, thus limiting the free H_2_O molecules and reducing the content of free H_2_O.^[^ ^30^, ^31^ ^]^ Therefore, the high ionic conductivity and excellent hydrophilicity of kaolin clay make it a prior choice for solid state electrolytes and separators in aqueous batteries.

Inspired by these unique merits, herein, we prepared a quasi‐solid‐state electrolyte by simply mixing kaolin clay and KOH solution for aqueous Al–air batteries. The abundant hydrophilic groups such as O─H, Si─O, and Al─O limit the free H_2_O molecules to achieve H_2_O‐poor environment on the Al/electrolyte interface and inhibit the HER. At the same time, the rapid ion channels are formed on the kaolin surface under the drive of the electric field due to the adsorption of cations in the electrolyte, which enhances the ionic conductivity of the electrolyte. Moreover, the closely contacted kaolin on the surface of Al anode can promote homogeneous Al ions stripping through the uniform pore size distribution of kaolin (Figure 1). Benefiting from these features, the corrosion rate of Al anode decreases from 0.075 mg cm^−2^ min^−1^ (Blank: 4 m KOH (4 mol L^−1^) solution) to 0.016 mg cm^−2^ min^−1^ (1:1 electrolyte: mass ratio of KOH solution to kaolin). And the quasi‐solid‐state electrolyte enables the Al–air full battery to achieve the ultra‐high mass‐specific capacity of 2765 mAh g^−1^ and excellent energy density of 4.56 KWh kg^−1^, which is highest among all the reported works. The quasi‐solid‐state electrolyte with low‐cost and simple production is valuable for large‐scale applications of Al–air batteries and provides a new strategy for other similar battery systems.

## 2 Results and Discussion

**Figure 2:**
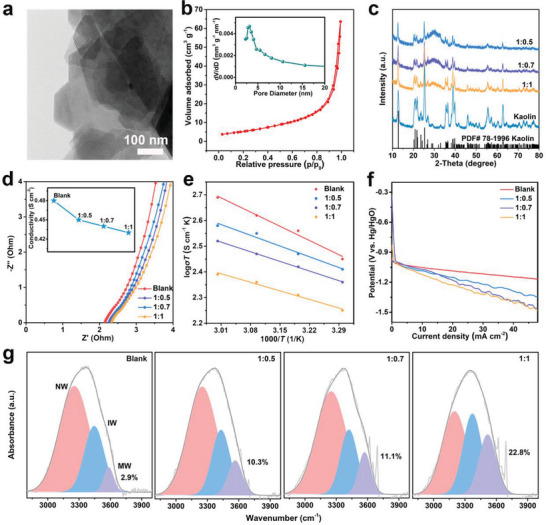
a) TEM image of pristine kaolin. b) Nitrogen adsorption/desorption isotherm plots and pore size distributions of kaolin. c) XRD patterns of pristine kaolin and different electrolytes. d) EIS spectra of different electrolytes at the high‐frequency region (Insert: the calculated ionic conductivities by EIS). e) Activity energy of different electrolytes. f) Polarization curves of different electrolytes. g) The fitted curves of different electrolytes represent three states of water molecules: NW, IW, and MW in FT‐IR spectra.

Kaolin with the general formula of Al_2_Si_2_O_5_(OH)_4_ is a 1:1 type layered silicate clay connected by Si─O tetrahedra and Al─O octahedra (Figure S1, Supporting Information).^[^ ^32^ ^]^ Scanning electron microscope (SEM) and transmission electron microscope (TEM) show the sheet structure of kaolin with the uniform distribution of Al, Si, O elements (**Figure** **2**a; Figure S2, Supporting Information). Brunauere–Emmette–Teller (BET) and Barrett–Joyner–Halenda (BJH) were performed to investigate the surface area and pore size of kaolin (Figure 2b). Kaolin has a specific surface area of 19.35 m^2^ g^−1^ and a typical mesoporous structure with a pore size of ≈3 nm was confirmed, which facilitates the rapid transport of metal ions in the channels. It can be seen that the kaolin contains 9.1 wt% crystal water and its structure remains stable even at 800 °C by thermogravimetric (TGA) analysis (Figure S3, Supporting Information).^[^ ^33^ ^]^ Then, we prepared quasi‐solid‐state electrolytes by simply mixing kaolin powder and 4 m KOH solution, as shown in Figure S4 (Supporting Information). When increasing the kaolin content, the electrolyte becomes more viscous until it turns into a quasi‐solid‐state. SEM images of kaolin in electrolytes are presented in Figure S5 (Supporting Information). As seen, the kaolin still maintains a sheet structure and X‐ray diffraction spectrometer (XRD) patterns of electrolytes in Figure 2c also shows high crystallinity compared to pure kaolin, which indicates the stability of kaolin in 4 m KOH solution. Figure S6 (Supporting Information) shows that the quasi‐solid‐state electrolytes possess similar electrochemical impedance spectroscopy (EIS) curves to the blank electrolyte. Encouragingly, the quasi‐solid‐state electrolyte shows a similar ionic conductivity to that of the blank electrolyte (0.48 S cm^−1^ for the blank electrolyte and 0.43 S cm^−1^ for the 1:1 electrolyte), which is mainly attributed to the abundant hydrophilic groups (O─H, Al─O, and Si─O) on the surface of kaolin, making it more soluble with the alkaline solution (Figure 2d). Moreover, the cations (K^+^) can be absorbed on the kaolin surface, and under the drive of electric field, cations will migrate through the weak adsorption sites similar to jump migration, thus forming a rapid ion channel on the kaolin surface and improving the ionic conductivity. In addition, the anions (OH^−^) will move in the opposite direction of the cations under the drive of electric field, which could decrease the interaction between the anions and cations, thus further improving the ionic conductivity in quasi‐solid‐state electrolyte.^[^ ^34^, ^35^ ^]^ As shown in Figure S7 (Supporting Information), all ionic conductivities show a similar upward trend as the temperature increases from 30 to 60 °C, and the quasi‐solid‐state electrolyte is only slight lower than the blank electrolyte. Furthermore, the activation energy is calculated by fitting the EIS results with Arrhenius equation. Interestingly, the 1:1 electrolyte has the lowest effective activation energy, indicating a reduced barrier for ion transmission (Figure 2e). These results furthermore confirm the existence of fast ion transport channels in quasi‐solid‐state electrolyte. In addition, the polarization curves are another key factor in evaluating the electrolyte performance. It can be seen that the potentials of quasi‐solid‐state electrolytes decrease only slightly with increasing current density at low current density (<10 mA cm^−2^), which are comparable to those of liquid blank electrolyte (Figure 2f). As expected, the quasi‐solid‐state electrolytes exhibit smaller polarization than previously reported liquid electrolytes (Figure S8, Supporting Information). As shown in Figure S9 (Supporting Information), there are no other impurity peaks in the fourier transform infrared (FT‐IR) spectra, which further implies the stability of kaolin in alkaline solution. In addition, the broad OH stretching can be fitted as three peaks located at ≈3208, ≈3412, and ≈3558 cm^−1^ by employing Gaussian function, corresponding to the network water (NW), intermediate water (IW) and multimer water (MW), respectively (Figure 2g).^[^ ^36^, ^37^ ^]^ Accordingly, the introduction of kaolin significantly decreases the content of NW and increases the content of MW by breaking the original hydrogen bond networks between H_2_O molecules due to the generation of new hydrogen bonds between H_2_O molecules and the hydrophilic groups on the kaolin surface. As a result, the activity of H_2_O molecules is reduced and contributes to the inhibition of Al anode corrosion.

**Figure 3:**
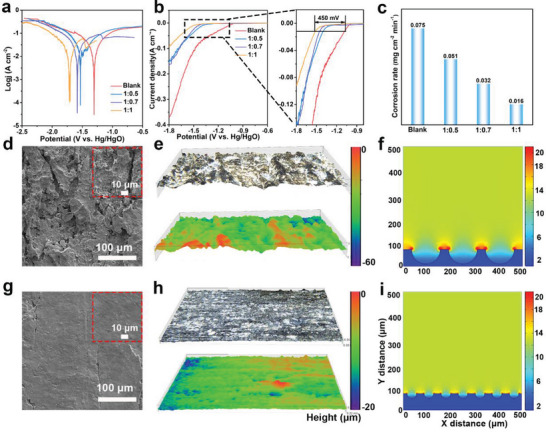
a) Tafel curves of different electrolytes. b) Electrochemical stability windows of different electrolytes. c) Corrosion rate of Al anode in different electrolytes. d) SEM images, e) in‐situ optical microscope images and f) simulated the electric field on surface of Al anode after immersed in blank electrolyte for 2 h. g) SEM images, h) in‐situ optical microscope images and i) simulated the electric field on surface of Al anode after immersed in 1:1 electrolyte for 2 h.

To explore the Al anode corrosion properties in different electrolytes, the potentiodynamic polarization curves were measured (**Figure** **3**a). The corrosion potential gradually shifts toward negative with the increase of kaolin content (−1.31 V for blank, −1.53 V for 1:0.5, −1.58 V for 1:0.7 and −1.71 V for 1:1 electrolytes), meaning that the self‐corrosion of Al anode is successfully suppressed. The electrochemical stability windows were further carried out to evaluate the HER potential in different electrolytes. It can be found that the HER potential increases from −1.07 V in blank electrolyte to −1.52 V in 1:1 electrolyte (10 mA cm^−2^), which indicates that the activity of H_2_O is reduced and a higher potential is needed to produce hydrogen gas in the quasi‐solid‐state electrolyte. These results are consistent with the results of the potentiodynamic polarization curves and further confirm that the self‐corrosion of Al anode in quasi‐solid‐state electrolyte is inhibited effectively. Additionally, the corrosion rate of Al anode in different electrolytes was investigated by the mass loss method. It can be seen that the Al anode has the highest corrosion rate (0.075 mg cm^−2^ min^−1^) in the blank electrolyte, which drops to 0.051, 0.032, and 0.016 mg cm^−2^ min^−1^ in 1:0.5, 1:0.7, and 1:1 electrolytes, respectively. Subsequently, the surface micromorphology of Al anodes after immersing in different electrolytes for 2 h were obtained by SEM and in‐situ optical microscope. The Al anode surface exhibits a loose, gully and rough structure compared to the smooth pristine Al surface after immersing in the blank electrolyte, indicating that the Al anode suffers from severe self‐corrosion in blank electrolyte (Figure S10, Supporting Information; Figure 3d). And this uneven structure will cause low anode utilization and unstable operating voltage during galvanostatic discharge process. Besides, the in‐situ optical microscope also reveals the uneven structure of Al surface, and the maximum corrosion depth reaches 60 µm (Figure 3e). Generally, this uneven corrosion strongly influences the electric field distribution at the electrode/electrolyte interface and a finite element simulation based on Comsol multiphysics was adopted to simulate the electric field distribution. As shown in Figure 3f, the electric field distribution is also uneven and a large amount of charge accumulates around the deep pits on the Al surface in blank electrolyte, which will aggravate corrosion along with deep pits. While the Al surface becomes more and more uniform with the addition of kaolin (Figure S11, Supporting Information; Figure 3g) and the corrosion deep also gradually decreases (40 µm for 1:0.5, 30 µm for 1:0.7, and 20 µm for 1:1), which is benefited from the uniform Al ion stripping and Al anode corrosion is successfully inhibited (Figure 3h). Moreover, the electric field distribution is more uniform on the Al surface in 1:1 electrolyte (Figure 3i). Therefore, the quasi‐solid‐state electrolyte not only inhibits the self‐corrosion of Al anode, but also promotes the uniform stripping of Al ions.

**Figure 4:**
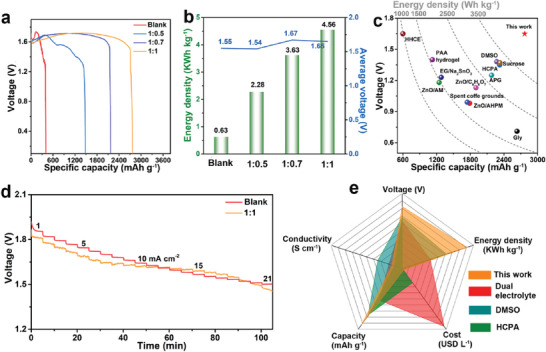
a) Galvanostatic discharge curves of full batteries with different electrolytes at a current density of 6 mA cm^−2^. b) Average voltage and energy density of different electrolytes. c) Comparison of specific capacities and voltages with reported electrolytes. d) Voltage of Al–air batteries at different current densities (Discharge for 5 min per current density). e) Comparison of some parameters in different electrolyte strategies.

To evaluate the electrochemical performance of Al anode in the quasi‐solid‐state electrolyte during the galvanostatic discharge process, the Al–air full batteries were constructed with commercial Al alloy and Mn_x_O_y_/Ag as anode material and cathode electrocatalyst, respectively. As shown in Table S2 (Supporting Information), the Al anodes show the higher anode utilization in the quasi‐solid‐state electrolyte after galvanostatic discharge for 3 h (39.35% for blank, 54.10% for 1:0.5, 92.93% for 1:0.7, and 96.68% for 1:1 electrolytes, respectively), further proving the inhibition effect of quasi‐solid‐state electrolyte on self‐corrosion of Al anode. It can be found that the mass‐specific capacity of Al–air battery with blank electrolyte is only 398 mAh g^−1^ at 6 mA cm^−2^, which is far away from the theoretical capacity (2980 mAh g^−1^), revealing that the Al anode suffered from severe self‐corrosion and only a small amount of energy was converted into electricity in the blank electrolyte. As for the quasi‐solid‐state electrolytes, the discharge capacity increases with the content increase of kaolin (1476 mAh g^−1^ for 1:0.5 and 2174 mAh g^−1^ for 1:0.7, respectively) (**Figure** **4**a; Table S3, Supporting Information). Especially, the full battery exhibits ultra‐high capacity of 2765 mAh g^−1^ with 1:1 electrolyte, closing to the theoretical specific capacity. In addition, a stable operating voltage supply is essential in practical applications. However, the discharge curve jitters seriously in the blank electrolyte, which can be explained that the disordered stripping of Al ions in the blank electrolyte and the formation and peeling of the discharge by‐product Al(OH)_3_ on the Al anode surface.^[^ ^19^, ^38^ ^]^ For the batteries with quasi‐solid‐state electrolytes, the discharge curves are smoother than that with blank electrolyte, suggesting the Al ions are uniform stripping (Figure S12, Supporting Information), which is in agreement with the results in Figure 3. In addition, kaolin powder still maintains the sheet and crystal structure after discharged, which means that kaolin is stable in the alkaline electrolyte and the result is consistent with Figure 2c (Figures S13 and S14, Supporting Information). Figure 4b shows that average operating voltages of Al–air batteries with quasi‐solid‐state electrolytes in galvanostatic discharge process are greater than blank electrolyte except for 1:0.5 electrolyte. The higher operating voltages indicate a faster ion migration rate in quasi‐solid‐state electrolytes, which further confirms the high ionic conductivity and lower activation energy of quasi‐solid‐state electrolytes. Accordingly, the batteries with quasi‐solid‐state electrolytes exhibit excellent energy density (Figure 4b), especially, the battery with 1:1 electrolyte can deliver an energy density of 4.56 KWh kg^−1^, highest among all the reported references (Figure 4c; Table S4, Supporting Information). Figure S15 (Supporting Information) shows the LSV curves of the pristine electrocatalyst and electrocatalyst after galvanostatic discharge in blank and 1:1 electrolytes. The electrocatalyst after galvanostatic discharge in 1:1 electrolytes exhibits higher diffusion limited current density and enhanced half‐wave potential than that in the blank electrolyte, indicating that the electrocatalyst in 1:1 quasi‐solid‐state electrolyte still possesses excellent catalytic activity and can be recycled after galvanostatic discharge. The reason for maintaining good catalytic performance can be attributed to the reduced contact of the alkaline solution with the electrocatalyst in the quasi‐solid‐state electrolyte, where the electrocatalyst suffers slight damage during discharge, and this can also explain the higher operating voltage of batteries with quasi‐solid‐state electrolyte in Figure 4b. Although battery polarization increases at high current density (15 mA cm^−2^) with quasi‐solid‐state electrolytes, the operating voltages still reach ≈1.4 V (Figure S16, Supporting Information), indicating that the quasi‐solid‐state electrolyte can meet the high power density requirements of devices. The operating voltages of Al–air batteries with blank and 1:1 electrolyte under different current density are shown in Figure 4d. It can be found that the voltages decrease only slightly with the increase of current density. Moreover, only one single battery with 1:1 quasi‐solid‐state electrolyte can power the light‐emitting diode (LED) screen (1–5 V), implying the practicability of Al–air batteries in suitable power devices (Figure S17, Supporting Information). Previously reported that high‐donor solvent (DMSO), “water‐in‐salt” electrolyte (HCPA) and gel‐KOH dual electrolyte strategies also have a beneficial effect on improving the Al–air battery performance.^[^ ^20^, ^21^, ^39^ ^]^ Some critical parameters for batteries with these three electrolytes are compared in Figure 4e. Clearly, as a kind of natural clay, kaolin is not toxic. At the same time, the quasi‐solid‐state electrolyte addresses the electrolyte leakage in the working process, thus possesses properties of environment friendliness and high safety. And the battery with this quasi‐solid‐state electrolyte delivers higher operating voltage and energy density than the reported dual electrolyte, DMSO and HCPA electrolyte.^[^ ^20^, ^21^, ^39^ ^]^ Most importantly, the low‐cost and simple fabrication of this kaolin‐based quasi‐solid‐state electrolyte can drive the larger‐scale application of Al–air batteries.

**Figure 5:**
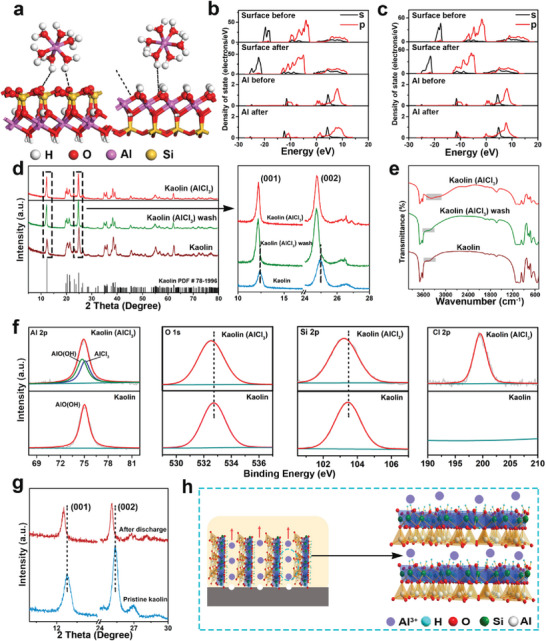
Adsorption configurations of [Al(H_2_O)_6_]^3+^ on a) Si─O surface (left) and Al─O surface (right). Partial density of state for Al with b) Si─O surface and c) Al─O surface. d) XRD patterns of pristine kaolin, kaolin (AlCl_3_) and kaolin (AlCl_3_) wash. e) FT‐IR spectra of pristine kaolin, kaolin (AlCl_3_) and kaolin (AlCl_3_) wash. f) High‐resolution XPS spectra for Al 2p, O 1s, Si 2p, and Cl 2p. g) XRD patterns of pristine kaolin and 1:1 quasi‐solid‐state electrolyte after discharge. h) Schematic diagram of Al ions transport in quasi‐solid‐state electrolyte.

In order to explore the mechanism of uniform stripping of Al ions in quasi‐solid‐state electrolyte, the density functional theory (DFT) and adsorption experiments were performed. According to the previous studies,^[^ ^40^ ^]^ the rational model of Al^3+^ is hydrated [Al(H_2_O)_6_]^3+^ in the water environment, and the hydroxylated Al─O and Si─O surfaces are exposed on each side (001) surface of kaolin. As shown in **Figure** **5**a, when [Al(H_2_O)_6_]^3+^ approaches the surface of Si─O and Al─O, the hydrogen bond (black dashed lines) between [Al(H_2_O)_6_]^3+^ and surface O atoms is the main interaction. Furthermore, the adsorption energy of [Al(H_2_O)_6_]^3+^ on Si─O surface and Al─O surface are −1.82 and −1.33 eV, respectively, which indicates that [Al(H_2_O)_6_]^3+^ will be preferentially adsorbed on the Si─O surface. Besides, the partial density of states of Al and Si─O surface were carried out to analyze the adsorption mechanism. Figure 5b shows that the density of state of Si─O surface shifts toward lower energy after adsorption and the delocalization of 2s and 2p orbitals are enhanced, suggesting the more stable state after adsorption of [Al(H_2_O)_6_]^3+^. Similarly, the density of state of Al also moves toward the lower energy direction, and the delocalization of Al enhances after adsorption, indicating the stability of Al. Additionally, similar density of state are observed after [Al(H_2_O)_6_]^3+^ absorbed on Al─O surface (Figure 5c).

Subsequently, the adsorption experiments were carried out to further investigate the interaction between Al ions and kaolin surface. 2 g kaolin was dispersed in 3 mL 2 m AlCl_3_ solution and stirred 24 h to obtain a uniform solution named as kaolin (AlCl_3_), in which the solvation configuration of the Al ion is [Al(H_2_O)_6_]^3+^. Figure 5d presents the XRD patterns of pristine kaolin, kaolin (AlCl_3_) and kaolin (AlCl_3_) after washing with water (kaolin (AlCl_3_) wash). The new diffraction peak located at 43.8°, which can be assigned as the characteristic peak of AlCl_3_, appears in the kaolin (AlCl_3_) and disappears after washing with deionized water (Figure S18, Supporting Information), confirming the interaction of Al ions and kaolin surface as electron attraction.^[^ ^41^ ^]^ In addition, the (001) and (002) crystal planes of kaolin are shift in the lower direction after the adsorption of AlCl_3_, and the expanded d‐spacing can be attributed to the adsorption of Al ions in the layer of kaolin. Moreover, it can be clearly found that OH stretching (3448 cm^−1^) and bending vibrational (1644 cm^−1^) of kaolin significantly widen with AlCl_3_ embedding into the kaolin surface in FT‐IR spectra (Figure 5e), suggesting the Al^3+^ may be adsorbed by OH groups and remain on the kaolin surface.^[^ ^42^ ^]^ Furthermore, X‐ray photoelectron spectroscopy (XPS) was employed to analyze the differences in chemical composition between kaolin and kaolin (AlCl_3_) (Figure S19, Supporting Information). Clearly, the full spectra and high‐resolution spectra show that the Al 2p, O 1s, and Si 2p have no obvious change after embedding AlCl_3_, meaning the structure of kaolin is stable after introducing AlCl_3_ (Figure 5f). Al 2p signal can be divided into two peaks at 74.9 eV and 75.5 eV, corresponding to AlO(OH) and AlCl_3_ in kaolin(AlCl_3_), respectively.^[^ ^43^ ^]^ While the Al 2p signal only exhibits an AlO(OH) peak in kaolin. In addition, the O 1s and Si 2p signals show similar offsets in kaolin(AlCl_3_) as a result of the interaction between kaolin and Al ions, consistent with the XRD results. To further verify the reaction of the actual discharge process in Al–air battery, the XRD of the quasi‐solid‐state electrolyte before and after discharge was performed. Figure 5g shows that the d‐spacing of (001) and (002) crystal planes of kaolin also expand after discharge process, which is similar to that in Figure 5d, indicating the adsorption of Al ions in the layer of kaolin during discharge process. Thus, both DFT calculations and spectroscopic analyze reveal that strong adsorption interactions exist between kaolin and Al ions and further confirm that Al ions are diffused through (001) and (002) crystal planes of kaolin after being stripped from the bulk Al anode, which promotes uniform stripping of Al ions in quasi‐solid‐state electrolyte (Figure 5h).

## 3 Conclusion

In summary, a novel quasi‐solid‐state electrolyte was constructed via a facile mixture of kaolin clay and alkaline solution to enhance the electrochemical performances of Al–air batteries. The abundance of hydrophilic groups on the kaolin surface not only restricted free H_2_O molecules through hydrogen bonds thus suppressing parasitic reaction, but also adsorbed metal cations in the solution, which favored both ionic conductivity and operating voltage. More importantly, the layer and uniform pore structure of kaolin promoted uniform stripping of Al ions and enhanced the stability of operating voltage. As a consequence, the as‐designed Al–air battery with quasi‐solid‐state electrolyte delivered ultra‐high mass‐specific capacity of 2765 mAh g^−1^ under a current density of 6 mA cm^−2^ and achieved the highest energy density of 4.56 KWh kg^−1^, 7.24 times higher than that with blank electrolyte. This facile and cost‐efficient quasi‐solid‐state electrolyte can facilitate the large‐scale commercial applications of Al–air batteries and provide valuable insights for other aqueous batteries.

## Conflict of Interest

The authors declare no conflict of interest.

## Supporting information

Supporting Information

## Data Availability

The data that support the findings of this study are available from the corresponding author upon reasonable request.
