# Supplementary material for: Quasi‐Solid‐State Aluminum–Air Batteries with Ultra‐high Energy Density and Uniform Aluminum Stripping Behavior
Source: Adv Sci (Weinh). 2023 Aug 16;10(29):2304214. doi: 10.1002/advs.202304214 (PMC10582464; doi:10.1002/advs.202304214)
Supplement: Supplementary file 1 — Supporting Information [file ADVS-10-2304214-s001.pdf]

ADVANCED SCIENCE
Open Access

Supporting Information

for *Adv. Sci.*, DOI 10.1002/advs.202304214

Quasi-Solid-State Aluminum–Air Batteries with Ultra-high Energy Density and Uniform Aluminum Stripping Behavior

Chaonan Lv, Yixin Li, Yuanxin Zhu, Yuxin Zhang, Jialin Kuang, Qing Zhao, Yougen Tang* and Haiyan Wang**

Quasi-Solid-State Aluminum-Air Batteries with Ultra-high Energy Density and Uniform Aluminum Stripping Behavior

Chaonan Lv, Yixin Li*, Yuanxin Zhu, Yuxin Zhang, Jialin Kuang, Qing Zhao, Yougen Tang* and Haiyan Wang*

C. Lv, Y. Li, Y. Zhu, Y. Zhang, J. Kuang, Y. Tang, H. Wang

Hunan Provincial Key Laboratory of Chemical Power Sources

College of Chemistry and Chemical Engineering, Central South University

Changsha, 410083, P. R. China.

E-mail: yixinli@csu.edu.cn (Yixin Li), ygtang@csu.edu.cn (Yougen Tang), wanghy419@csu.edu.cn (Haiyan Wang)

Q. Zhao

Key Laboratory of Advanced Energy Materials Chemistry (Ministry of Education),

College of Chemistry, Nankai University

Tianjin, 300071, P. R. China

Experimental Section

Electrode preparation: The aluminum alloy used in this work was purchased from Changzhou Yoteco, and its elemental composition is shown in Table S1. The aluminum alloy was mechanically polished with sandpaper, then washed with deionized water and absolute ethanol in an ultrasonic cleaner for 5 min, respectively, and then dried in a vacuum at 70 °C for 8 h to obtain the anode material. The air cathode is commercially available which is composed of a Mn_xO_y@Ag catalyst, gas diffusion layer and Ni mesh current collector.

Electrolyte preparation: Potassium hydroxide was of the analytical grade from Shanghai Chemical Reagent Company of China. Kaolin was purchased from Aladdin.

By mixing different mass of 4 M KOH solution with kaolin powder, 1:0.5, 1:0.7 and 1:1 electrolytes were prepared, while 4 M KOH solution was marked as Blank.

Characterization: The morphology and composition of the aluminum surface were systematically investigated by using a scanning electron microscope (SEM, Nova Nano-SEM 230), in-situ optical microscope (Nikon, SMZ25) and transmission electron microscope (TEM, JEM-2100F). Fourier transform infrared (FT-IR) spectra were obtained using a Bruker Vertex 70 FT-IR spectrophotometer. The Brunauere–Emmette–Teller (BET) specific surface areas of the kaolin were determined by micromeritics ASAP 2460 and the pore size distributions were obtained by the Barrett–Joyner–Halenda (BJH) model. X-ray photoelectron spectroscopy (XPS) measurements were performed on ESCALAB 250 Xi X-ray photoelectron spectrometer (Thermo Fisher). X-ray diffraction spectrometer (XRD) patterns were performed on Shimadzu XRD-6000.

Electrochemical measurements: Electrochemical measurement was carried out in a conventional three-electrode system by using a CHI760 electrochemical workstation, which used an aluminum alloy (10 mm × 10 mm × 3 mm) as the working electrode (WE), a Hg/HgO electrode as the reference electrode (RE) and platinum as the counter electrode (CE). The potentiodynamic polarization curves were obtained from 0.5 V to 1.5 V vs. the open circuit potential (OCP). The electrochemical impedance spectroscopy (EIS) experiments were performed at the OCP in the frequency from 100 kHz to 0.01 Hz with 5 mV amplitude. The electrochemical windows of the different electrolytes were obtained by linear sweep voltammetry (LSV) at 1 mV s^{-1} in the three-

electrode system.

Aluminum–air full battery tests: The aluminum-air full batteries were composed of an aluminum plate anode, electrolyte and two cathode films with a Mn_xO_y@Ag catalyst. The mass-specific capacity of the full battery was calculated by dividing the mass difference of the aluminum anode before and after galvanostatic discharge.

Computational details: All calculations were performed by the Material Studio 2019 software. Adsorption energy (E_{ad}) was performed by the CASTEP, and the exchange-correlation energy was approximately described by the Perdew-Burke-Ernzerhof (PBE) functional based on the generalized gradient approximation (GGA). The cutoff energy of the plane wave base was set in 400 eV and the surface of kaolin (001) was optimized with a 20 Å vacuum layer. The adsorption energy between molecules A and B is described as:

The adsorption energy between molecules A and B is described as:

$$E_{ad} = E_{AB}-E_{A}-E_{B} \tag{1}$$

E_{AB} is the energy of $[Al(H_2O)_6]^{3+}$ adsorbed on the surface of kaolin (001) surface (Si-O or Al-O surface). E_A is the energy of $[Al(H_2O)_6]^{3+}$, E_B is the energy of Si-O or Al-O surface.

Electric filed simulation: In order to simulate the electric field distribution at the interface between anode and electrolyte, a Finite Element Analysis (FEA) model was performed using COMSOL Multiphysics 5.4 software with the "Tertiary Current Distribution". The size of the entire two-dimensional model was set to 500 μm×500 μm. The deep pit of aluminum surface was represented by three semicircles with a radius

60 μm while the distance between them was 155 μm for blank electrolyte, as for 1:1 electrolyte, the deep pits was represented by seven semicircles with a radius 20 μm and the distance between them was 72 μm. It should be noted that the sizes in these simulations are based on the in-situ optical microscope images. In addition, a transient simulation of the process was performed in an area filled with electrolyte.

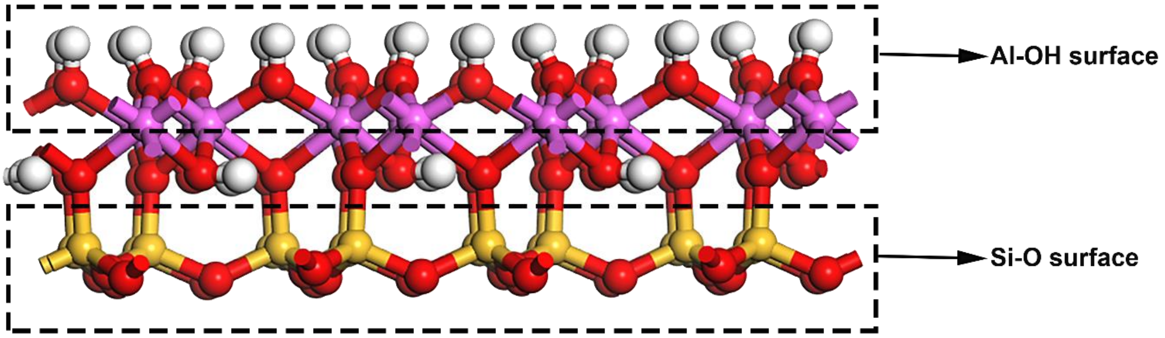

Fig. S1. Crystal structure of kaolin powder.

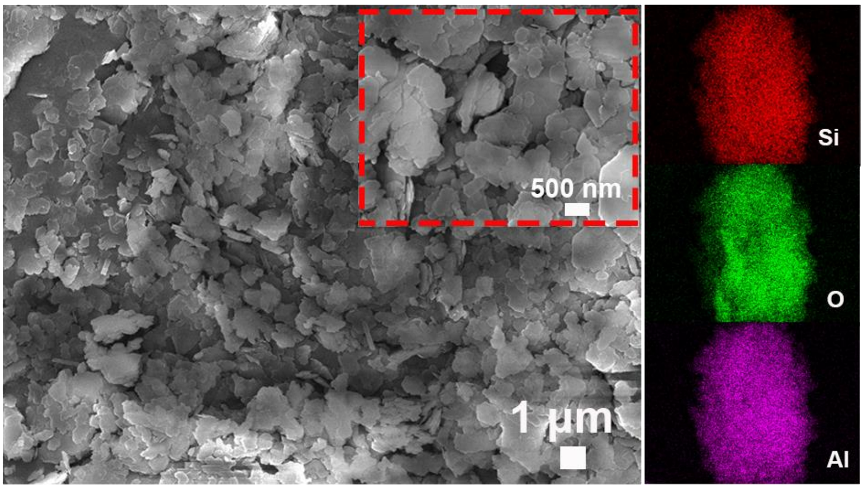

Fig. S2. SEM and mapping images of pristine kaolin powder.

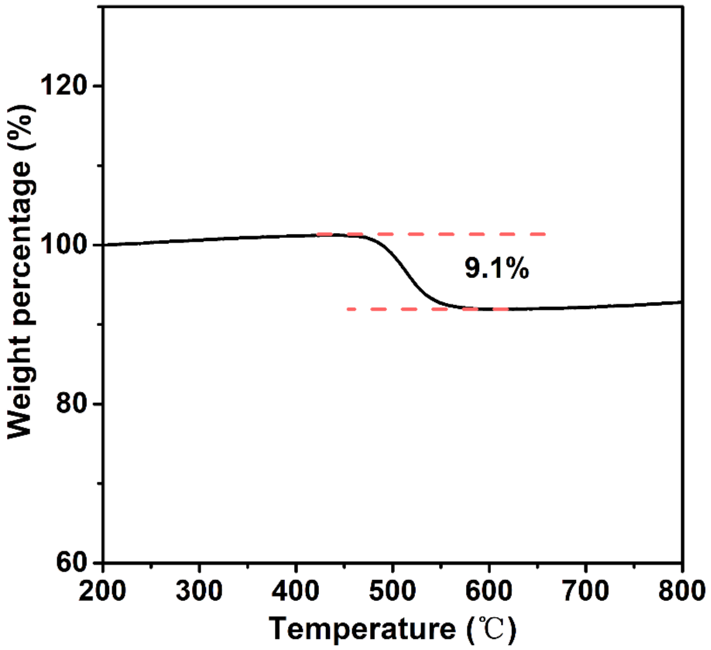

Fig. S3. Thermogravimetric curve of kaolin powder.

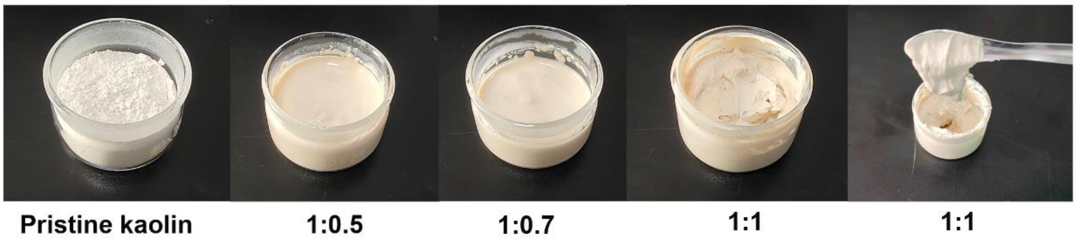

Fig. S4. Digital photograph of pristine kaolin powder and quasi-solid electrolytes.

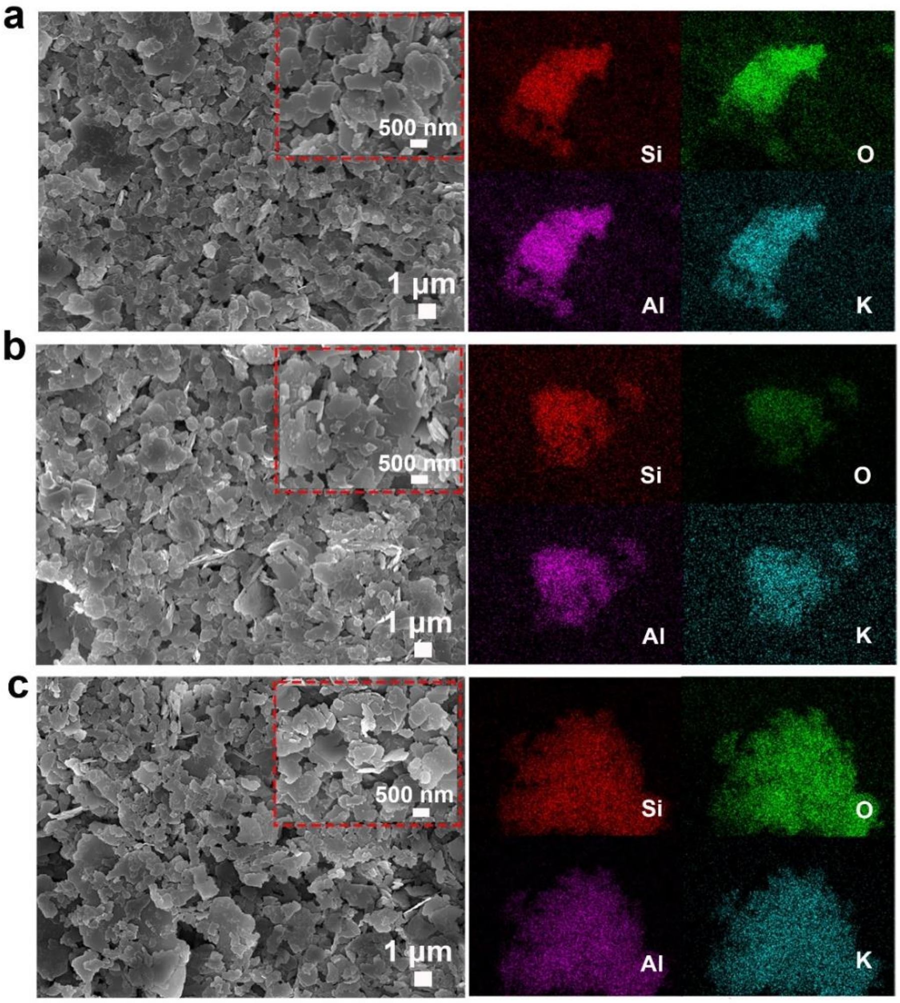

Fig. S5. SEM and mapping images of (a) 1:0.5, (b) 1:0.7 and (c) 1:1 electrolytes.

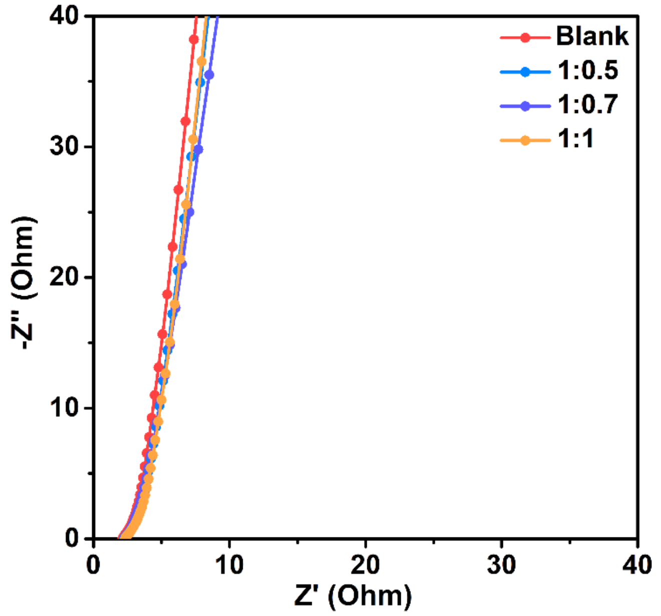

Fig. S6. EIS spectra results of different electrolytes at 25 °C.

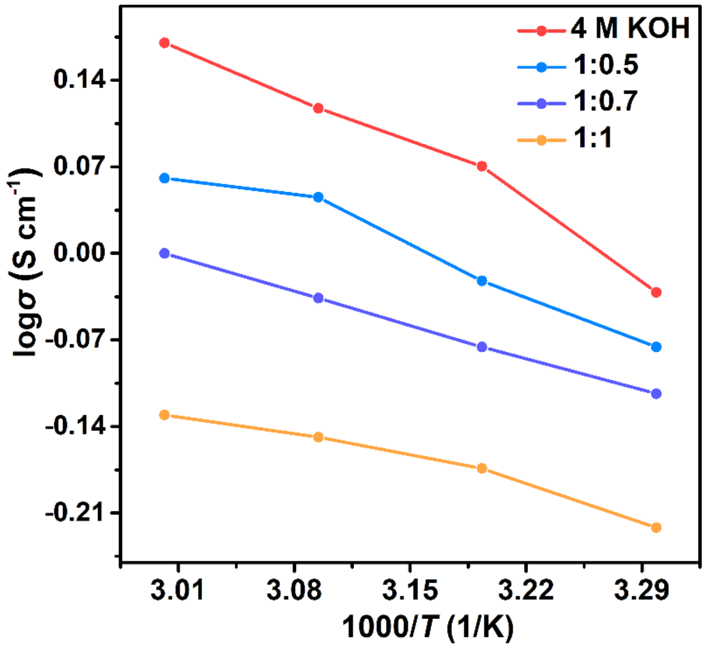

Fig. S7. Ionic conductivity of different electrolytes under different temperatures.

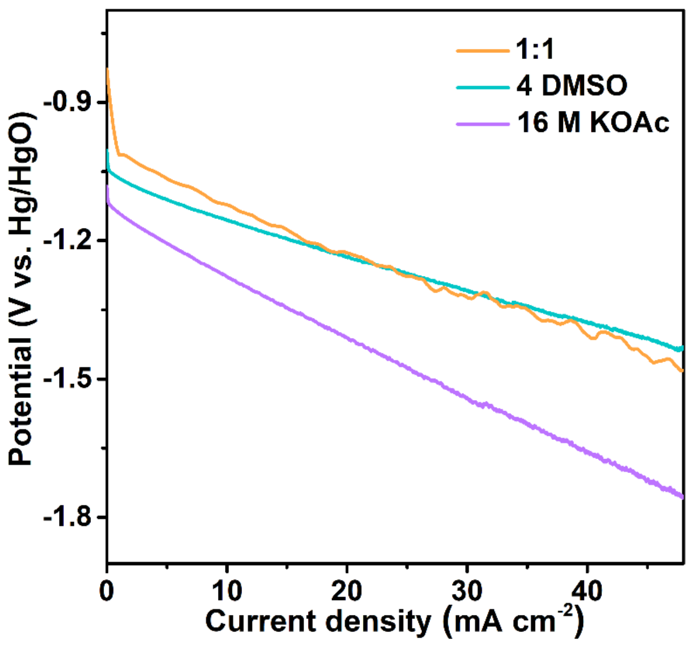

Fig. S8. Polarization curves of different electrolytes. (4 DMSO: 40 vol% of DMSO and 4 M NaOH solution. 16 M KOAc: 16 mol kg^{-1} potassium acetate in 4 M KOH solution).

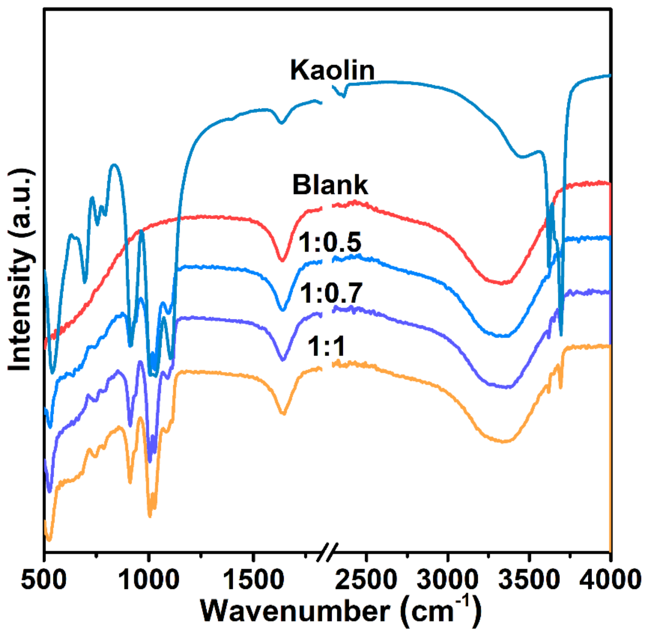

Fig. S9. FT-IR spectra of different electrolytes and pristine kaolin powder.

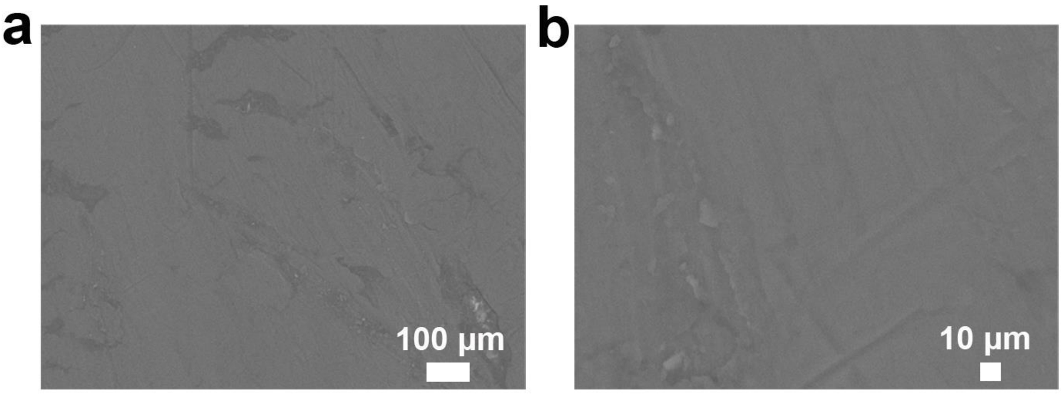

Fig. S10. SEM images of the pristine Al anode.

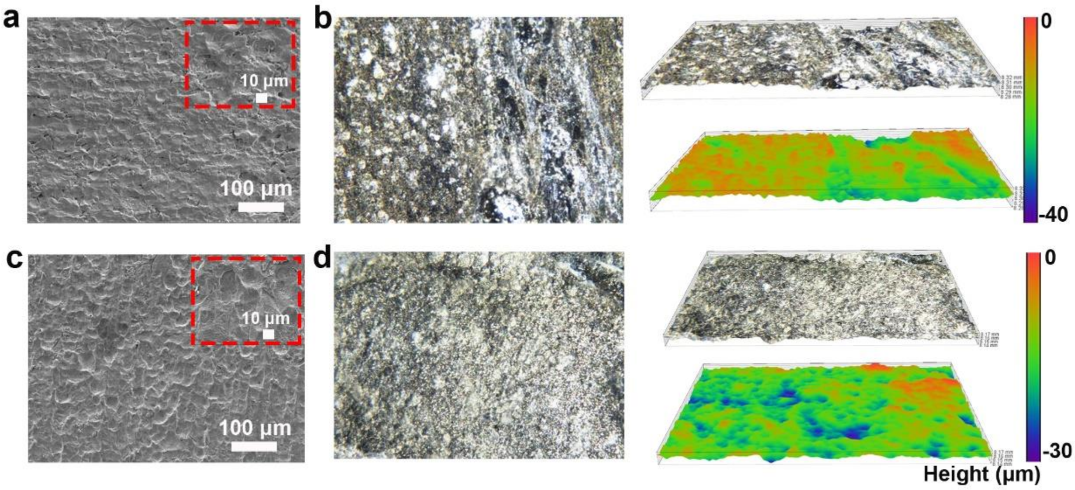

Fig. S11. (a-b) SEM images and in-situ optical microscope images of Al anodes immersed in 1:0.5 and (d-e) 1:0.7 electrolytes for 2 h.

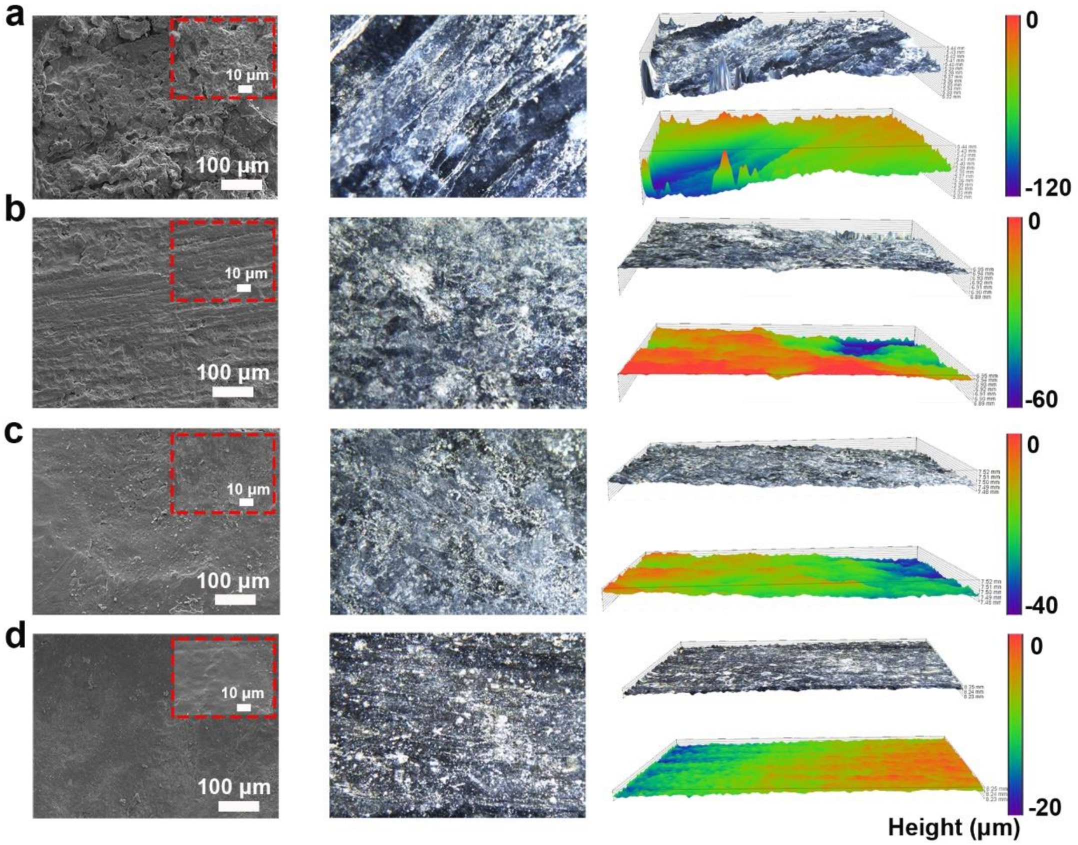

Fig. S12. In-situ optical microscope images of Al anode surfaces in (a) blank, (b)1:0.5, (c) 1:0.7 and (d) 1:1 electrolytes after galvanostatic discharge.

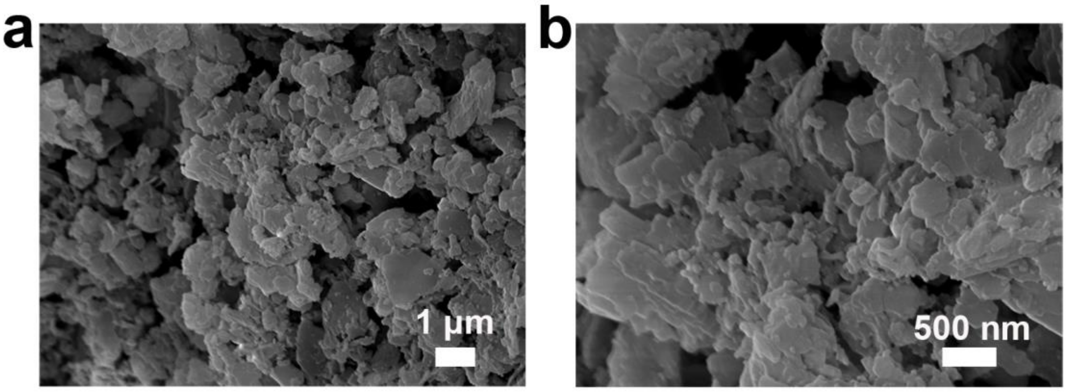

Fig. S13. SEM images of kaolin powder after discharge.

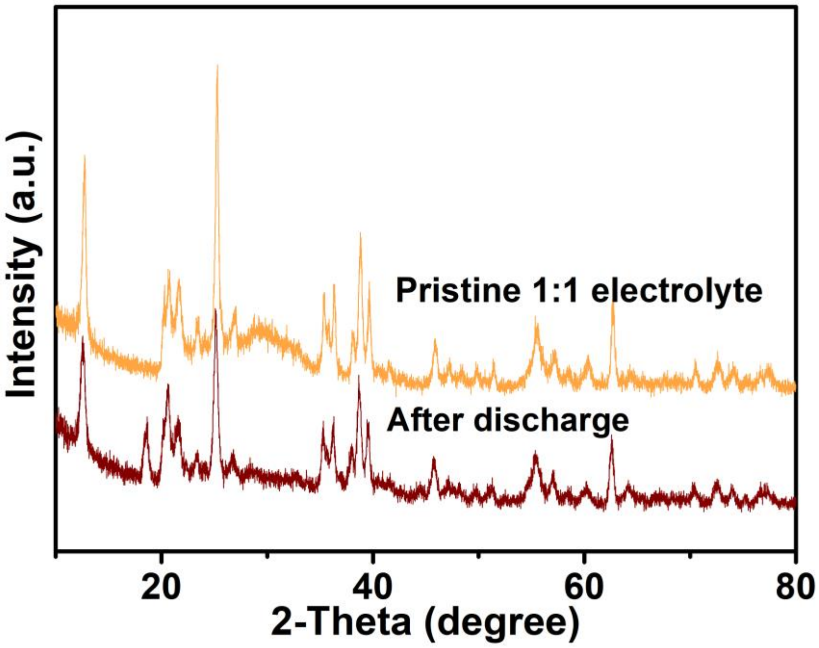

Fig. S14 XRD patterns of pristine 1:1 quasi-solid-state electrolyte and after discharged.

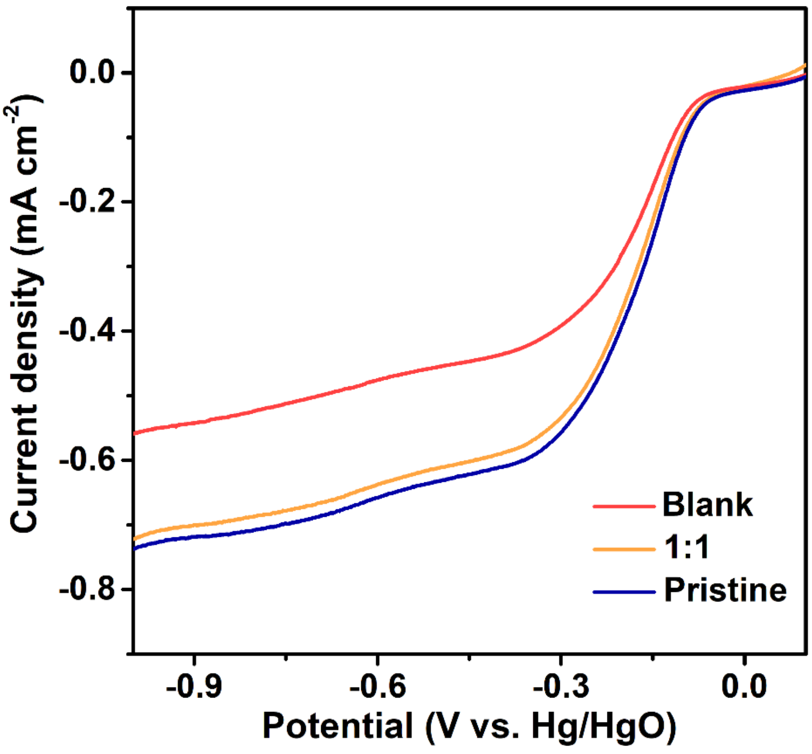

Fig. S15. LSV curves (1600 rpm) of electrocatalysts in different electrolytes after galvanostatic discharge in 4 M KOH solution.

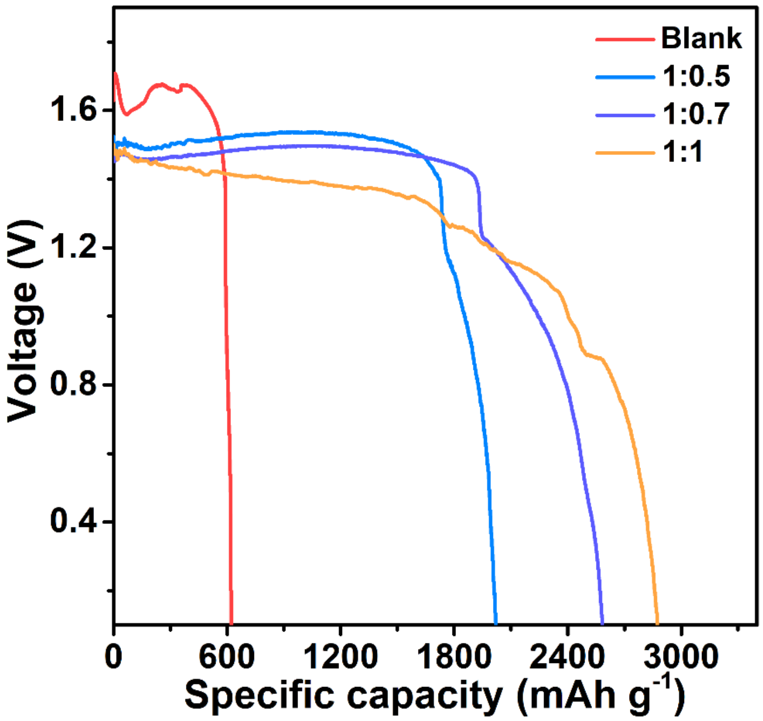

Fig. S16. Galvanostatic discharge curve of full batteries with different electrolytes at a current density of 15 mA cm^{-2}

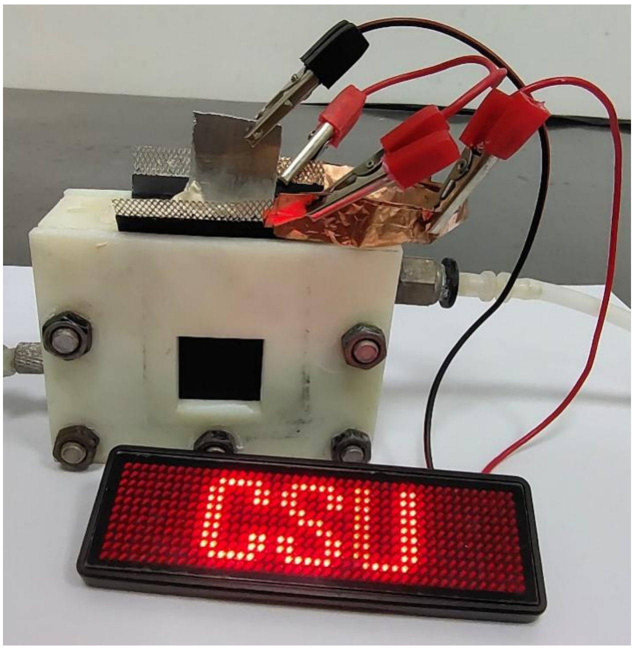

Fig. S17. LED light powered by Al-air battery with 1:1 quasi-solid electrolyte.

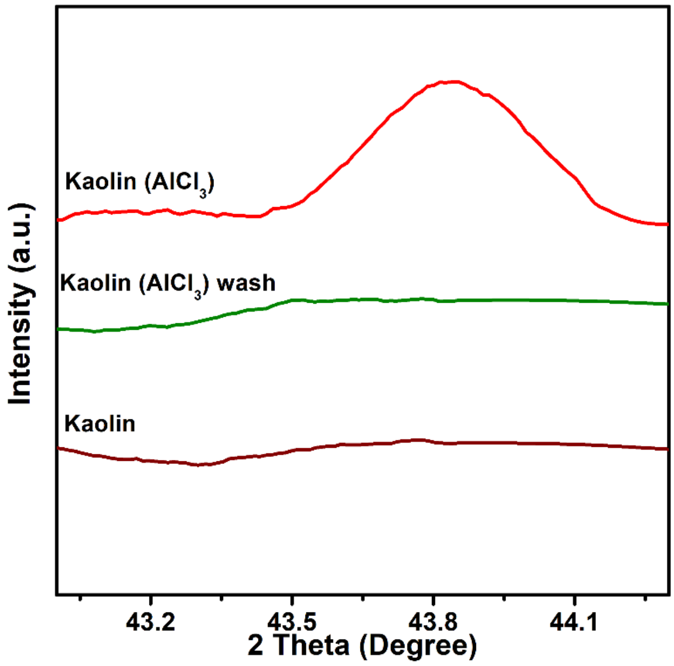

Fig. S18. XRD patterns of pristine kaolin, kaolin ($AlCl_3$) and kaolin ($AlCl_3$) wash samples.

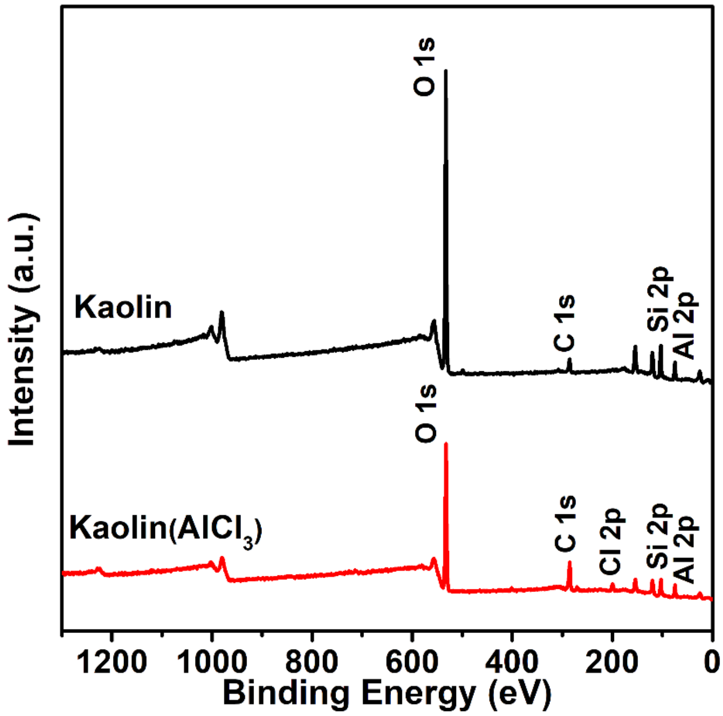

Fig. S19. XPS full spectra of pristine kaolin and kaolin ($AlCl_3$) samples.

Table S1 Chemical compositions of Al alloy (wt %)

Mg	Ga	Sn	Zn	Fe	Cu	Si	Al
0.024	0.011	0.010	0.004	≤0.009	≤0.001	≤0.001	Remainder

Table S2 Al anode utilization in different electrolytes after galvanostatic discharge for 3 h.

Electrolyte	ΔM (g)	U_a%
Blank	0.128	39.35
1:0.5	0.0931	54.10
1:0.7	0.0542	92.93
1:1	0.0521	96.68

Table S3 Aluminum anode mass consumption before and after discharge.

Electrolyte	△M (g)	Specific capacity (mAh g^{-1})
Blank	4.7169	398
1:0.5	0.3027	1476
1:0.7	0.2218	2174
1:1	0.1303	2765

Table S4. Comparison of various electrolyte additives based on the consumed mass of Al anode materials.

Electrolyte additives	Current density [mA cm^{-2}]	Specific capacity [mAh g^{-1}]	Voltage [V]	References
1:1 Quasi-solid electrolyte	20	2765	1.65	This work
$ZnO/C_6H_8O_7$	10	1902	1.13	Ref. [1]
PAA hydrogel	10	1126	1.4	Ref. [2]
ZnO/AM	25	1246.0	1.18	Ref. [3]
Spent coffee grounds	20	1746.72	0.99	Ref. [4]
EG/Na_2SnO_3	5	1282.9	1.23	Ref. [5]
HHCE	5	600	1.65	Ref. [6]
DMSO	25	2271.39	1.38	Ref. [7]
Gly	10	2632	0.71	Ref. [8]
AHMP/ZnO	20	1789	0.981 V	Ref. [9]
Sucrose	10	2330	1.37	Ref. [10]
APG/ Na_2SnO_3	100	2180	1.25	Ref. [11]
HCPA	25	2324	1.35	Ref. [12]

References:

[1] H. Jiang, S. Yu, W. Z. Li, Y. H. Yang, L. S. Yang, Z. J. Zhang, *J. Power Sources* **2020**, *448*, 227460.

[2] S. G. Zhang, Y. C. Wang, Y. W. Li, M. H. Wei, K. L. Wang, *J. Power Sources* **2022,** 545, 231907.

[3] H. Cheng, T. Wang, Z. Li, C. Guo, J. Q. Lai, Z. L. Tian, *ACS Appl. Mater. Interfaces* 2021, 13, 51726−51735.

[4] W.-H. Lee, S.-R. Choi, J.-G. Kim, *ACS Omega* **2021**, *6*, 25529−25538.

[5] C. C. Ma, C. Q. Hu, X. B. Xu, Y. Song, M. Y. Shao, J. C. Lin, Z. C. Jiang, *ChemistrySelect* **2021**, *6*, 1804 –1813.

[6] C. N. Lv, Y. X. Zhang, J. J. Ma, Y. X. Zhu, D. Huang, Y. X. Li, H. Y. Wang, Y. G. Tang, *J. Mater. Chem. A* **2022**, *10*, 9506–9514.

[7] T. Wang, Z. Tian, Z. You, Z. Li, H. Cheng, W. Li, Y. Yang, Y. Zhou, Q. Zhong, Y. Lai, *Energy Storage Mater*. **2022**, *45*, 24-32.

[8] T. H. Pham, W.-H. Lee, J.-H. Byun, J.-G. Kim, *Energy Storage Mater.* **2023**, *55*, 406–416.

[9] L. Luo, C. Zhu, L. J. Yan, L. Guo, Y. Zhou, B. Xiang, *Chem. Eng. J.* **2022***, 450,* 138175.

[10] P. F. Wu, Q. Zhao, H. S. Yu, Z. Tang, Y. H. Li, D. Huang, D. Sun, H. Y. Wang, Y. G. Tang, *Chem. Eng. J.* **2022**, *438*, 135538.

[11] S. A. Wu, Q. Zhang, D, Sun, J. Y. Luan, H. W. Shi, S. Y. Hu, Y. G. Tang, H. Y. Wang, *Chem. Eng. J.* **2020,** *383*, 123162.

[12] S. A. Wu, S. Y. Hu, Q. Zhang, D. Sun, P. F. Wu, Y. G. Tang, H. Y. Wang, *Energy Storage Mater.* **2020**, *31*, 310–317.
